# Genetic selection for growth drives differences in intestinal microbiota composition and parasite disease resistance in gilthead sea bream

**DOI:** 10.1186/s40168-020-00922-w

**Published:** 2020-11-23

**Authors:** M. Carla Piazzon, Fernando Naya-Català, Erick Perera, Oswaldo Palenzuela, Ariadna Sitjà-Bobadilla, Jaume Pérez-Sánchez

**Affiliations:** 1 Fish Pathology Group, Instituto de Acuicultura Torre de la Sal (IATS-CSIC), Castellón, Spain; 2 Nutrigenomics and Fish Endocrinology Group, Instituto de Acuicultura Torre de la Sal (IATS-CSIC), Castellón, Spain

**Keywords:** *Sparus aurata*, Selective breeding, Growth, Plant-based diets, Genome-diet interactions, Intestinal bacteria, *Enteromyxum leei*, Domestication, Plasticity

## Abstract

**Abstract:**

**Background:**

The key effects of intestinal microbiota in animal health have led to an increasing interest in manipulating these bacterial populations to improve animal welfare. The aquaculture sector is no exception and in the last years, many studies have described these populations in different fish species. However, this is not an easy task, as intestinal microbiota is composed of very dynamic populations that are influenced by different factors, such as diet, environment, host age, and genetics. In the current study, we aimed to determine whether the genetic background of gilthead sea bream (*Sparus aurata*) influences the intestinal microbial composition, how these bacterial populations are modulated by dietary changes, and the effect of selection by growth on intestinal disease resistance. To that aim, three different groups of five families of gilthead sea bream that were selected during two generations for fast, intermediate, or slow growth (F3 generation) were kept together in the same open-flow tanks and fed a control or a well-balanced plant-based diet during 9 months. Six animals per family and dietary treatment were sacrificed and the adherent bacteria from the anterior intestinal portion were sequenced. In parallel, fish of the fast- and slow-growth groups were infected with the intestinal parasite *Enteromyxum leei* and the disease signs, prevalence, intensity, and parasite abundance were evaluated.

**Results:**

No differences were detected in alpha diversity indexes among families, and the core bacterial architecture was the prototypical composition of gilthead sea bream intestinal microbiota, indicating no dysbiosis in any of the groups. The plant-based diet significantly changed the microbiota in the intermediate- and slow-growth families, with a much lower effect on the fast-growth group. Interestingly, the smaller changes detected in the fast-growth families potentially accounted for more changes at the metabolic level when compared with the other families. Upon parasitic infection, the fast-growth group showed significantly lower disease signs and parasite intensity and abundance than the slow-growth animals.

**Conclusions:**

These results show a clear genome-metagenome interaction indicating that the fast-growth families harbor a microbiota that is more flexible upon dietary changes. These animals also showed a better ability to cope with intestinal infections.

Video Abstract

## Background

Aquaculture is the fastest growing food production sector and already produces more than 50% of the fish for human consumption [1]. Fish, as a source of high-quality protein and many essential micronutrients, is a key product for food security and good nutrition in both developed and developing countries, and its importance grows with the increasing human population. Fish arise as one of the most sustainable animal protein production sectors because of its high feed conversion efficiency, its high food quality, and its lower carbon footprint when compared with other animal production systems [2]. However, still many efforts are being conducted to improve the sustainability of the aquaculture sector to support its fast growth.

One of the main issues in aquaculture production is the use of marine resources, mainly derived from fisheries, as the main protein and oil ingredients in aquafeed. Due to the stagnation of the catches and the increased demand for both human food and aquafeed, great efforts are being conducted to introduce alternative and more sustainable raw materials [3, 4]. High replacement levels of fish meal and fish oil with plant materials were successful in terms of growth [5–10], food safety [11], fillet texture, shelf life, and sensory freshness [12, 13]. However, plant-based diets have shown some drawbacks. For instance, intestinal pro-inflammatory profiles, loss of integrity and functionality of the intestinal epithelium, disease susceptibility, or changes in the sex ratio have been described when carnivorous fish were fed substitution diets [10, 14–18]. Nevertheless, most of these problems could be solved by formulating more balanced diets and supplementing them with different additives, like sodium butyrate [10, 18, 19].

Selective breeding to improve growth rates and disease resistance has been documented in aquaculture for more than a century [20–24]. Large scale family-based breeding programs are now established as the industry standard for genetic improvement of aquaculture species. The success of these programs in fish is explained by the relatively high heritability for economically important traits, the high fecundity, and short generation intervals [21]. In gilthead sea bream (*Sparus aurata*), the species of interest in this study, and the main farmed fish in the Mediterranean [25], genetic selection has been applied to improve growth rates, feed conversion, mortality rates, skeletal deformities, disease resistance, fillet yield and flesh and carcass quality [26–30]. We have recently demonstrated that selection for faster growth in gilthead sea bream is associated with a more continuous growth across the season and a high level of intestinal plasticity. Briefly, fast-growth families demonstrated the ability of reshaping their intestines to maximize nutrient absorption when fed plant-based diets, i.e., they had shorter intestines when fed standard diets, but they exhibited longer intestines when fed plant-based diets. This intestinal plasticity was genetically regulated and correlated with changes in liver and adipose tissue and fast growth rates both in favorable (summer) and non-favorable (winter) seasons [31]. The intestinal plasticity of this protandrous hermaphrodite fish has also been reported previously, with increased villi length and a larger number of goblet cells in fish fed plant-based diets [32, 33]. This plasticity allows gilthead sea bream to adapt to dietary changes with no impact on growth or health. However, none of these previous studies focused on the changes induced in the intestinal microbiota.

Intestinal microbiota is key for many host functions, such as digestion, nutrient absorption and metabolism, disease resistance, and immune training and function. Its importance in health has led to an increasing interest in manipulating these populations to improve animal welfare, not only in humans but also in livestock and fish [34]. Due to its economic value, many studies on gilthead sea bream intestinal microbiota have been conducted. These studies were mainly focused on defining baseline populations [35–37] or changes induced by diet [18, 38–44] or environmental conditions [45]. The microbiota is composed of very dynamic populations that are affected by different factors [18, 36, 42, 46–55] such as diet, season, habitat, rearing density, age, sex, and genetic background, the focus of the current study. There are not many studies defining the effects of the host genome on intestinal microbiota composition in fish. However, it has been demonstrated that there is a clear correlation between fish genotype and intestinal microbial communities [56–60]. Yet, to date, there is no information on the genome × intestinal microbiota interaction of genetically selected gilthead sea bream and how this can affect diet plasticity, health, and disease resistance.

This study had three objectives. To evaluate whether the genetic background of three different groups of gilthead sea bream families (five families in total), selected for slow, intermediate, and fast growth, affects the resident bacterial microbiota of the anterior intestine, the segment where nutrient absorption takes place mainly. To determine how these microbial communities are shaped upon feeding with a well-balanced plant-based diet. And lastly, to study the influence of the different genotypes on disease resistance, using infection with an intestinal parasite of the economic impact on gilthead sea bream aquaculture. Overall, this study aimed to determine the selection for heritable growth × metagenome interaction and the effect on diet plasticity and disease susceptibility to help improve intestinal health of gilthead sea bream in aquaculture.

## Results

### Samples and sequencing results

Five different gilthead sea bream families selected by growth derived from the PROGENSA (Spanish selection program of gilthead sea bream) broodstock were used in this study (F3 generation) [27, 28, 31]. The five families were then grouped in three sets, named suprafamilies, by their different growth trajectories as previously described [31]: fast (families e5e2 and e6e2, constituting suprafamily e5e6), intermediate (family c2c7), and slow (families c4c2 and e4e1, constituting suprafamily c4e4). It is important to bear in mind that these families are not of clonal origin, they have substantial genomic heterogeneity, and were grouped considering phenotypic characters. These animals fed a control (D1) or a well-balanced plant-based diet (D2) for 9 months, were kept in a common garden in order to eliminate the disturbing effects that could appear by rearing the analyzed families in different tanks. Six fish of each family and diet were used to sample the adherent microbiota of the anterior intestine. In total, 60 samples were sequenced: 24 samples for the fast- and slow-growth suprafamilies and 12 samples for the intermediate-growth group. More details on the rearing of the fish and sampling can be found in the methods section.

After Illumina sequencing of the 60 samples, three samples were eliminated from further analysis due to low quality of the reads (one from suprafamily c4e4 fed with D1 and two from suprafamily e5e6 fed with D1). The remaining 57 samples yielded 8,803,202 high quality reads, with a mean of 154,442 reads per sample, ranging from 99,040 to 303,044 (Additional file 1: Table S1). The reads were assigned to 2327 OTUs at 97% identity threshold. Almost half of these OTUs (49.8%) were classified up to the level of species, 89.3% to the level of genus and more than 95% to the levels of family (95.1%), order (97.5%), class (98.8%), and phylum (99.9%). Rarefaction analysis showed curves that approximated saturation (horizontal asymptote); thus, a good coverage of the bacterial community was achieved and the number of sequences for analysis was considered appropriate (Additional file 2: Figure S1).

### Microbiota diversity and composition

**Fig. 1.**
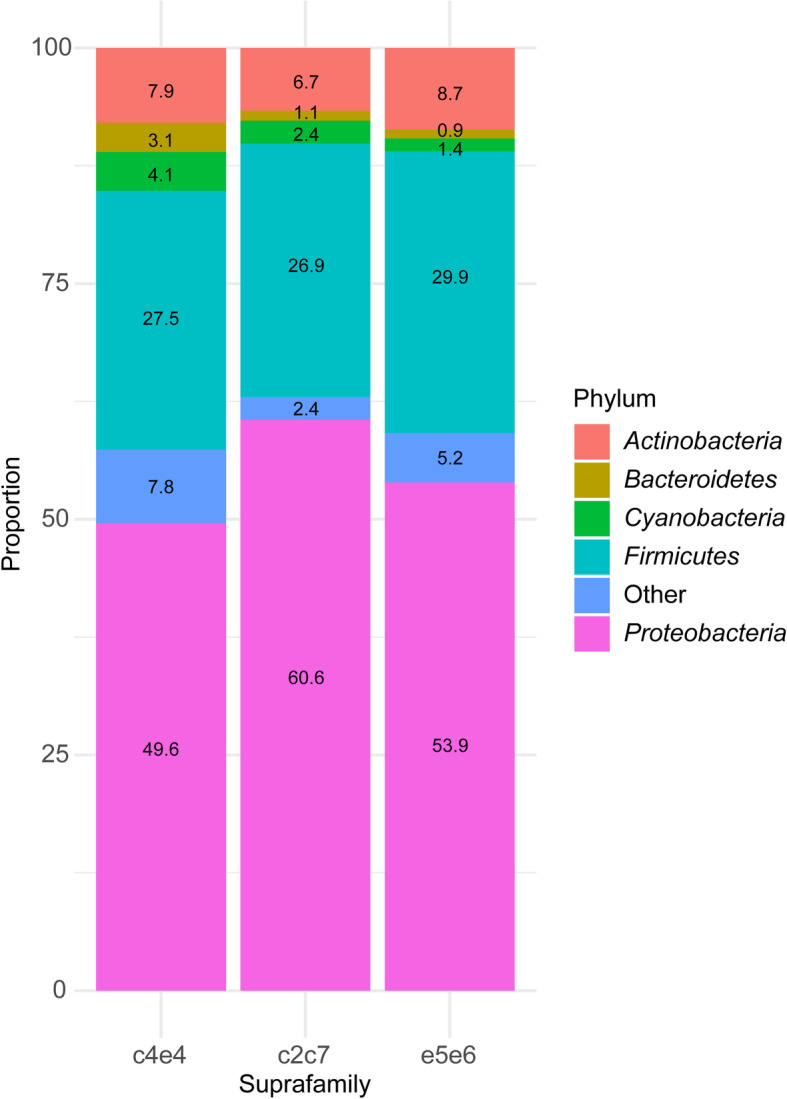
Stacked bar chart representing the relative abundance of bacterial phyla in the different groups of families (c4e4, c2c7, and e5e6). No significant differences were found in the abundance of the different phyla among groups (Kruskal-Wallis test)

When comparing the bacterial diversity and composition of the three groups of families, regardless of the diet, no significant differences were found in richness (Chao1 and ACE) and diversity (Shannon and Simpson) indexes (Table 1). The overall bacterial composition at the phylum level was also not different among groups (Kruskal-Wallis test, Dunn’s post-test), indicating that all these animals harbor the typical microbiota expected in gilthead sea bream intestines (Fig. 1). In all groups of families, *Proteobacteria* was the most abundant phylum, constituting ≥50% of the total resident bacteria in the anterior intestine. *Vibrionaceae* and *Oceanospirillaceae* families were the most abundant *Proteobacteria* in all groups, representing 18.9 and 11.6%, 19.5 and 14.5%, and 22.3 and 8.2% of the bacterial communities from e5e6, c2c7, and c4e4, respectively (Additional file 3: Figure S2). The second most abundant phylum was *Firmicutes* (≥27% in all groups), with the families *Clostridiaceae*, *Listeriaceae*, and *Staphylococcaceae* representing ~20% of the total microbiota in all groups. The phylum *Actinobacteria* (≥7%, particularly families *Corynebacteriaceae*, *Micrococcaceae*, and *Propionibacteriaceae*), followed by *Cyanobacteria* (≥1.5%) and *Bacteroidetes* (≥1%), were also abundant in the three groups (Additional file 3: Figure S2).

**Table 1:** Species richness estimates (Chao1 and ACE) and diversity indexes (Shannon and Simpson)

	Suprafamily			K-W test
	e5e6	c2c7	c4e4	***P*** value
Chao1	713.5 ± 177.4	1221 ± 411.5	960.6 ± 321.3	0.153
ACE	449.5 ± 37.46	415.6 ± 19.49	362.9 ± 15.24	0.155
Shannon	2.785 ± 0.12	2.9 ± 0.16	2.807 ± 0.09	0.682
Simpson	0.85 ± 0.03	0.884 ± 0.03	0.877 ± 0.02	0.454

Richness and diversity estimates of the suprafamilies e5e6 (*n* = 22), c2c7 (*n* = 12), and c4e4 (*n* = 23) are represented as mean ± SEM. No statistical differences were found among groups (K-W test: Kruskal-Wallis test)

### Diet and family effects on microbiota

**Fig. 2:**
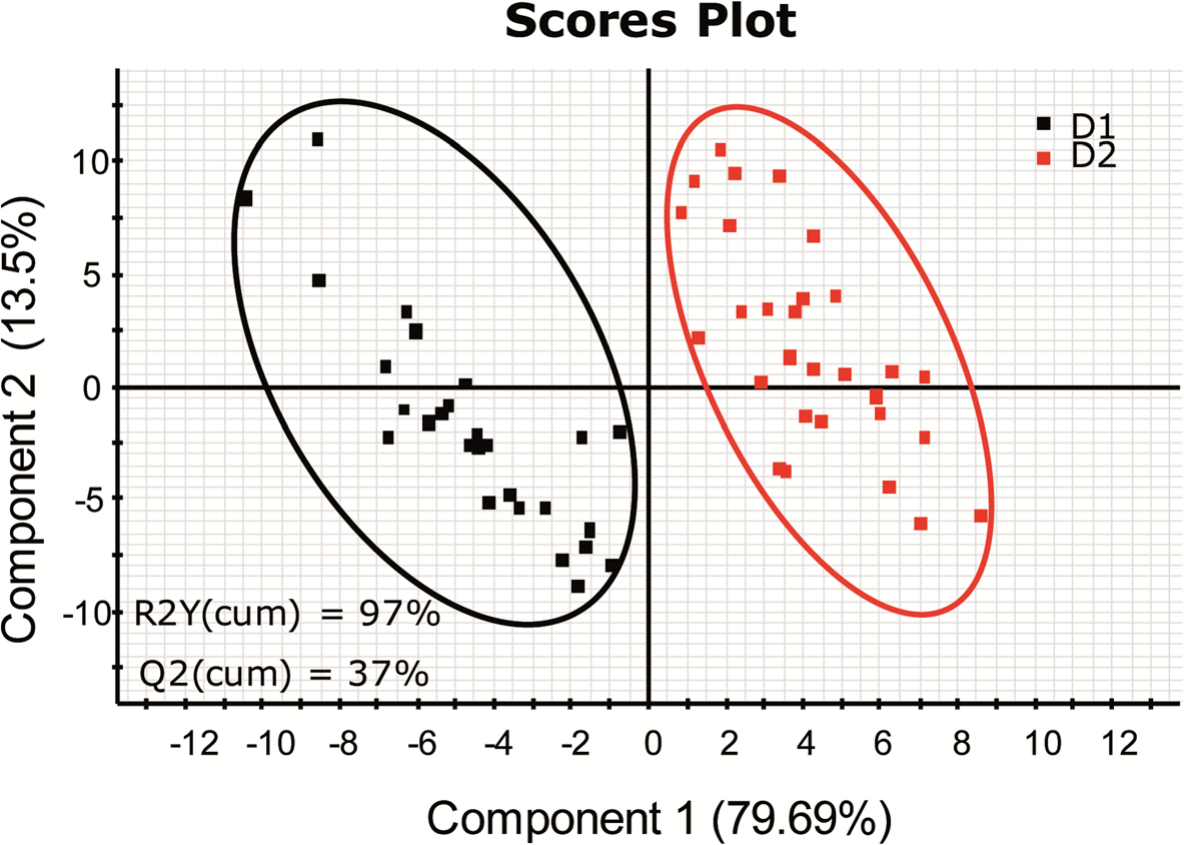
Diet changes intestinal mucus microbiota. Two-dimensional PLS-DA score plot was constructed using the variable diet representing the distribution of the samples between the first two components in the model. The goodness of fit and validation by the permutation test can be found in Additional file 4: Figure S3A and B

A PERMANOVA test was used to study differences in bacterial composition by diet, family, and the interaction diet × family. Not taking into account the diet, the family variable showed no statistically significant effect on the microbiota (*P* = 0.169, *F* = 1.308, *R*^2^ = 0.044). However, statistical differences were detected when comparing animals fed different diets, regardless of the genetic background (*P* = 0.041, *F* = 1.913, *R*^2^ = 0.032). Although *R*^2^ values detected were quite low, they were in line with what was reported in other microbiota studies [61]. This is due to the complexity and variability of microbiota samples. To corroborate and study in more detail these differences, a PLS-DA model was constructed and statistically validated. In Fig. 2, the PLS-DA model shows a clear separation of fish fed D1 or D2 along with component 1 (79.69%). The PLS-DA model was successfully validated with a permutation test discarding the possibility of over-fitting of the supervised model (Additional file 4: Figure S3A and B). These results highlight that the diet has a significant impact on the composition of the adherent bacterial communities of the anterior intestine, masking differences among families.

**Fig. 3:**
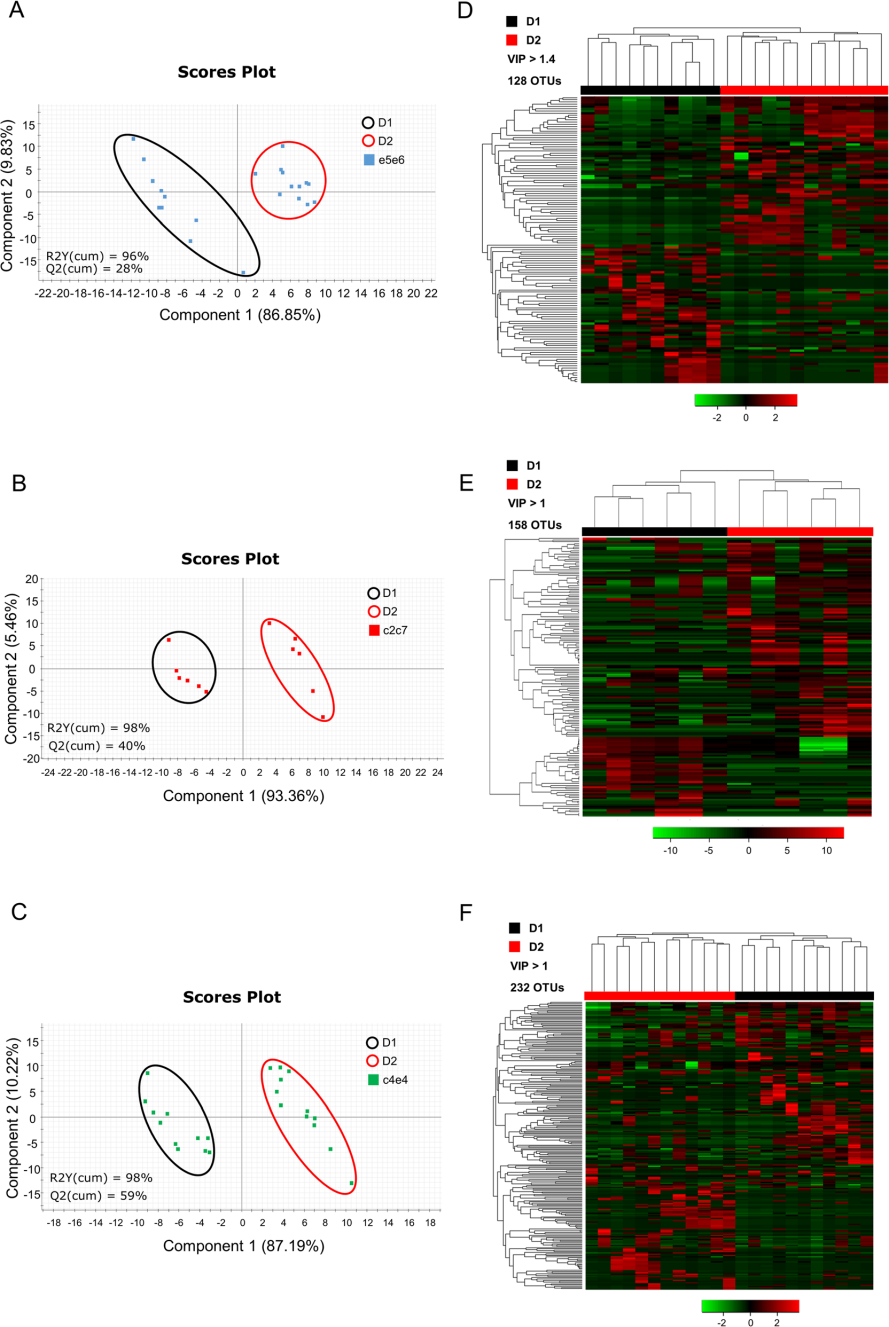
Microbiota changes induced by diet depend on the genetic background. Two-dimensional PLS-DA score plots constructed using the variable diet in the fast- (**a**), intermediate- (**b**), and slow-growth (**c**) families separately, representing the distribution of the samples between the first two components in the model. The goodness of fit and validation by the permutation test can be found in Additional file 4: Figure S3C–H. Heatmaps represent the abundance distribution (*Z-*score) of the OTUs identified to be driving the separation by diet in fast- (**d**), intermediate- (**e),** and slow-growth (**f**) families. Control (D1) and plant-based diets (D2) are represented in black and red, respectively

The PERMANOVA results of the interaction showed that significant differences appeared when taking into account both, genetic background (suprafamilies) and diet (*P* = 0.017, *F* = 1.843, *R*^2^ = 0.062). The PLS-DA models of the different suprafamilies showed a separation of fish fed D1 from those fed D2 (Fig. 3a, b, and c). The permutation tests (Additional file 4: Figure S3 C–H) showed that only the models for c2c7 and c4e4 could be validated, while the model for e5e6 was over-fitted. This means that significant differences in bacterial communities due to diet were mainly occurring in the groups of families with intermediate (c2c7) and slow (c4e4) growth. By contrast, differences in the fast-growth group were not statistically significant.

To determine which groups of bacteria were driving these separations with the diet changes, a more detailed analysis of the variable importance in projection (VIP) was performed throughout a heatmap representation. Hierarchical clustering of samples was applied and the minimum VIP values significantly driving the separation of the groups in the model were calculated, with the OTUs within these values being selected for further analysis. Differences in diet were mainly changing 128 OTUs (VIP >1.4) in suprafamily e5e6, 158 OTUs (VIP >1) in family c2c7 and 232 OTUs (VIP >1) in suprafamily c4e4 (Fig. 3d, e, and f). A detailed list of the VIPs can be found in Additional file 5: Table S2.

### Microbiota changes due to diet depend on the genetic background

**Fig. 4:**
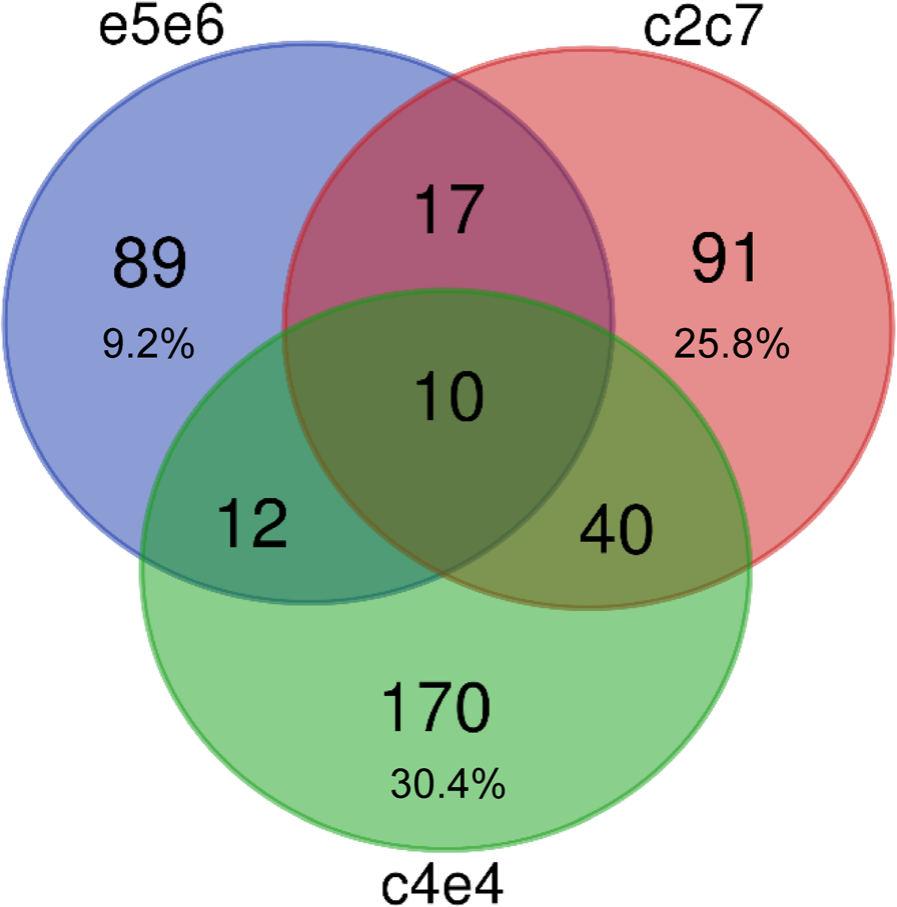
Most of the OTUs driving the separation by diet are family exclusive. Venn diagram depicting unique and shared OTUs responsible for the separation by diet in the different groups of families. The percentages refer to the proportion relative to the overall microbiota that the unique OTUs constitute in each suprafamily

A detailed study of the different OTUs driving the separation due to diet in the different suprafamilies revealed that most were family exclusive, and only 10 of these OTUs were changing in all groups (Fig. 4). The number of OTUs exclusively changing in groups e5e6, c2c7, and c4e4 were 89, 91, and 170, respectively. The greatest difference was found when studying the abundance of these exclusive OTUs in the different groups. Exclusive OTUs changing in groups c2c7 and c4e4 accounted for 25.8 and 30.4%, respectively, of the total bacterial composition. Interestingly, the 89 OTUs changing in group e5e6 only accounted for 9.2% of the total bacterial communities in these animals.

**Fig. 5:**
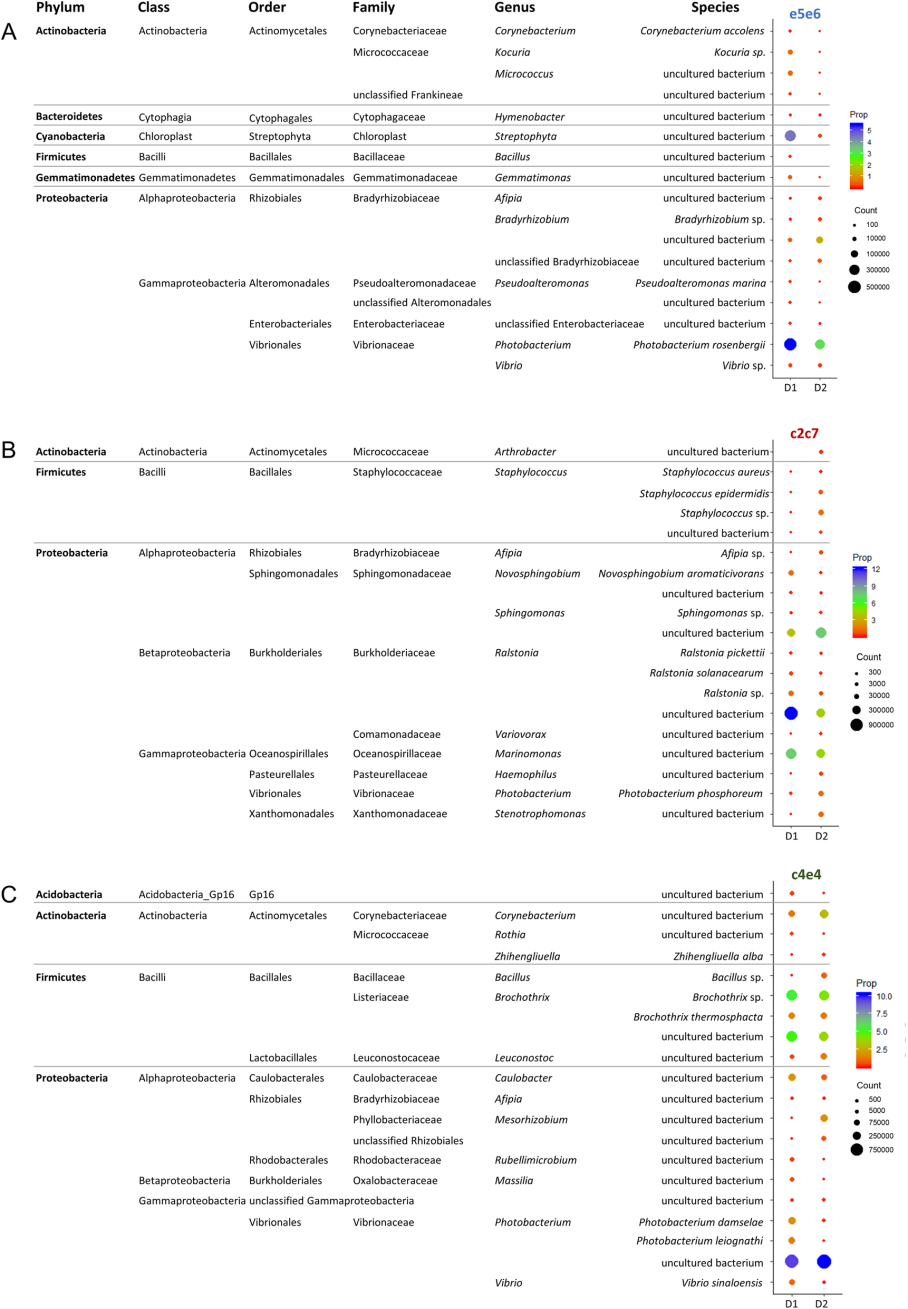
Dot plot map depicting the most abundant OTUs that exclusively drive the separation by diet in the fast- (**a**), intermediate- (**b**), and slow-growth (**c**) families. The size of the dots represents the normalized counts in each group (D1, control diet; D2, plant-based diet). The color scale represents the mean abundance, in percentage, of each OTU within each group of family and diet

Figure 5 shows the most abundant bacteria of those that exclusively drive the separation by diet in the different groups of families. In suprafamily e5e6, *Actinobacteria* (particularly *Kokuria* and *Micrococcus*) and *Cyanobacteria* decreased and some *Firmicutes* (*Bacillus* sp.) disappeared when animals were fed D2. Regarding *Proteobacteria*, some genera increased (A*fipia, Bradyrhizobium*) and others decreased (*Pseudoalteromonas, Photobacterium, Vibrio*) with the plant-based diet (Fig. 5a). In the intermediate growth family (c2c7), some *Actinobacteria* (*Arthrobacter* sp.) appeared, and different species of the genus *Staphylococcus* (*Firmicutes*) increased with the D2 diet. *Proteobacteria* again appeared more variable, with *Novosphingobium*, *Ralstonia,* and *Marinomonas* decreasing, and *Sphingomonas*, *Haemophilus*, *Photobacterium*, and *Stenotrophomonas* increasing with D2 (Fig. 5b). In suprafamily c4e4, the *Actinobacteria* genera *Corynebacterium* and *Zhihengliuella,* and the *Firmicutes Bacillus* increased, while the *Firmicutes* genera *Brochothrix* decreased with D2. Within *Proteobacteria*, the genera *Caulobacter*, *Vibrio,* and some *Photobacterium* (*P. damselae* and *P. leiognathi*) decreased and *Mesorhizobium* and other *Photobacterium* sp. increased with D2 (Fig. 5c).

### Predicted metabolic differences are larger in groups with fewer bacteria changes

**Fig. 6:**
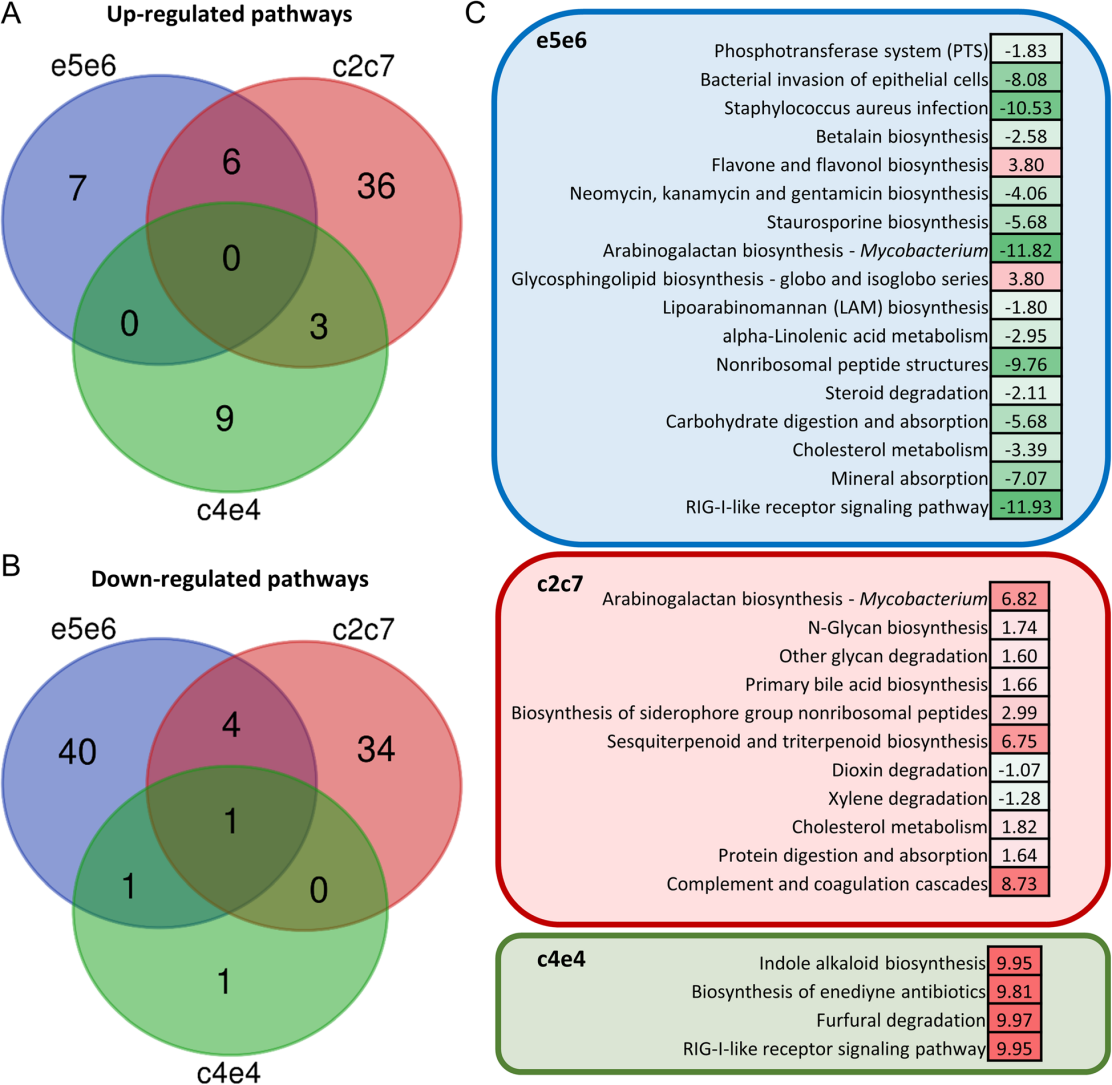
Changes in diet differentially affect the metabolic capacity of each group of families. Venn diagrams depicting unique and shared pathways detected to be up- (**a**) or down-regulated (**b**) by diet in the different groups of families. The most significantly represented pathways (log2 fold change > |1|) in each group of families are shown in (**c**). The numbers are the log2 fold change of the pathway when comparing D2 versus D1. Red and green shades are used to show degrees of up- and down-regulation, respectively

In an attempt to evaluate the biological significance of the differences induced by diets in the microbiota of the different groups of families, pathway analysis was performed using the inferred metagenomes of the OTUs driving the separation by diet. The results showed that in e5e6 (fast growth), 59 pathways could be significantly changing with the different diets. Whereas in c2c7 (intermediate growth), 84 pathways showed differences, and only 15 pathways were predicted to be changing in the slow-growth group (c4e4) (Additional file 6: Table S3). Very few overlaps were found when comparing the pathways predicted to be changing in each group of families when fed D2 in comparison to D1. In group c2c7, the number of over- and under-represented pathways was balanced, whereas most of the changing pathways in group e5e6 were under-represented, and in group c4e4 were over-represented in D2 (Fig. 6a and b). Among the most under-represented pathways in e5e6, there were several pathways related to infection, inflammation, or activation of the immune system (bacterial invasion of epithelial cells, *Staphylococcus aureus* infection, Arabinogalactan biosynthesis or RIG-I-like receptor signaling pathway). The only highly over-represented pathways (logFC >1) were Flavone and flavonol biosynthesis and Glycosphingolipid biosynthesis (Fig. 6c). In group c2c7, most of the highly differentially abundant pathways (logFC > |1|) were over-represented, highlighting Arabinogalactan biosynthesis, Sesquiterpenoid and triterpenoid biosynthesis and complement and coagulation cascades. In c4e4, very few pathways were differentially abundant, with only 4 pathways with logFC > |1|, which were highly over-represented: Indole alkaloid biosynthesis, biosynthesis of enediyne antibiotics, Furfural degradation and RIG-I like receptor signaling pathway (Fig. 6c). Of note, these results were obtained using prediction software optimized for mammalian samples; thus, they only reflect the metabolic potential of these populations.

### Genetic background effects on disease resistance

**Fig. 7:**
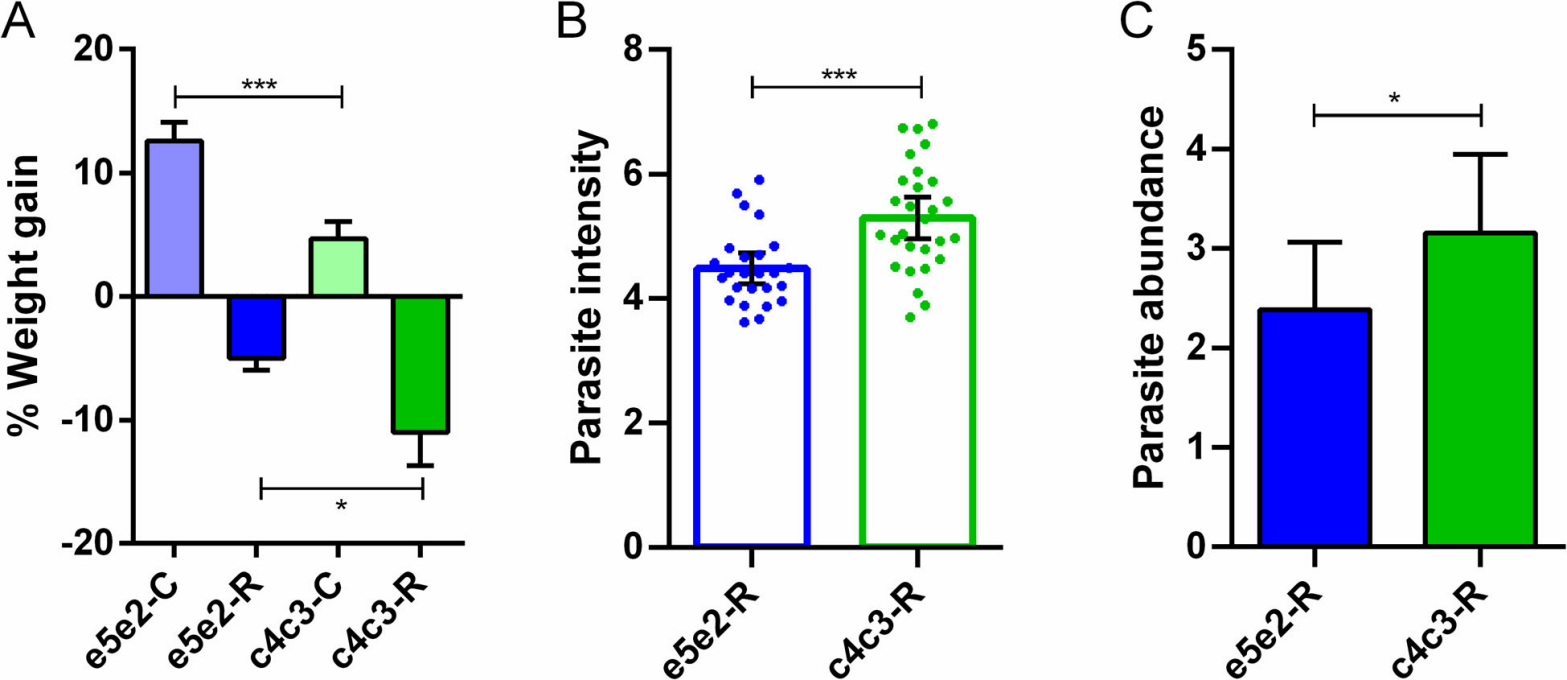
Slow-growth families are more susceptible to enteric parasite infection. **a** Difference in weight (in percentage) of control (C) or *Enteromyxum leei* infected (R) fish after 70 days post-infection relative to weight at day 0. Data are represented as mean + SEM. Differences between families were tested by Student’s *t* test (control groups *P* = 0.0003, infected groups *P* = 0.0331). **b** Intensity of infection represented as mean + 95% CI of log transformed parasite DNA copies per fish. Each dot represents the intensity value of an infected individual. Differences between families were tested by Student’s *t* test (*P* = 0.0003). **c** Parasite abundance represented as mean + 95% CI of log transformed parasite DNA copies per fish. Differences between families were tested by Mann-Whitney test (*P* = 0.031) and Kolmogorov-Smirnov test (*P* = 0.017)

Taking into account the differences detected in intestinal morphology, plasticity, and microbial composition between fish selected for fast and slow growth, the different response upon an intestinal pathogen was also evaluated. Two families were selected for this study, e5e2 (fast growth) and c4c3 (slow growth). These animals were kept together in the same tanks, fed D2 diet and challenged with the intestinal myxozoan parasite *Enteromyxum leei*. This parasite lives and divides in the intestinal epithelium inducing intestinal damage, impaired nutrient absorption and anorexia. Therefore, among the most common disease signs induced by this parasite are diminished growth and weight loss [62]. After 70 days, post-infection (dpi) biometric parameters showed that, as expected, the weight gain, relative to the weight at 0 dpi, of the control uninfected fish was significantly higher in the fast-growth family (12.6%) than in the low-growth group (4.7%). Upon infection, the fast-growth family showed a significantly lower weight loss (5%) than the-slow growth group (11%) (Fig. 7a). The prevalence of infection was 78 and 87% for groups e5e2 and c4c3, respectively. Moreover, the fast-growth group showed the significantly lower intensity of infection and parasite abundance values when compared with c4c3, the low-growth family (Fig. 7b and c).

## Discussion

Intestinal microbiota is composed of very diverse and dynamic microbial communities that can be affected by many factors, with environment, diet, and host genetics among the most important [63, 64]. In the current study, the intestinal adherent bacterial communities of three different groups of families of gilthead sea bream selected by growth and fed two different diets were studied and compared. The animals were kept together in the same tanks and conditions in order to eliminate the environmental variable. The results showed that the genetic background of gilthead sea bream influences the structure of the commensal bacterial communities and their fluctuations upon differences in diet. These changes can have important metabolic implications and impact disease resistance.

Breeding programs in aquaculture are mainly focused on the genetic gain in growth rate and feed efficiency and evaluate these productive traits at harvest [21, 22]. In addition, selection to improve disease resilience has also become a highly desirable breeding goal and has been studied against different pathogens including parasites [65, 66]. The heritability of different traits linked to growth selection has also been documented. For instance, genetic correlations between growth rate and disease resistance or survival have been described [67, 68]. In gilthead sea bream, in particular, productive traits linked to growth selection, such as mortality rates, disease resistance, tissue biometrics, intestinal plasticity, skeletal deformities, fillet yield, and flesh and carcass quality, have been evaluated [26–31, 69]. However, the relation between intestinal microbiota and growth selection has never been studied in this species. Intestinal microbiota is considered an “extra organ” that plays key roles in overall and intestinal development, physiology, growth, and health. It regulates feeding, digestive and metabolic processes, and immune response [64, 70]. This prompts the “chicken or the egg” question, that is: Is the selection for genes related to growth constituting the genetic background that will select the resident bacteria? Or are we selecting for certain bacterial configurations that will shape the growth trajectories of the animals? Probably both are true, but at the moment, we still do not have answers to those questions. Nonetheless, our results clearly show that there is a relationship among gilthead sea bream genetic background, diet, and the microbial communities living in their intestines.

The current results showed that the adherent bacterial communities of the anterior intestine of the different families of gilthead sea bream fed two different diets do not differ significantly in their overall core composition. As in previous studies with this species, the phyla *Proteobacteria*, *Firmicutes*, *Actinobacteria*, and *Bacteroidetes* dominate the autochthonous microbiota of the intestine [36, 41, 43]. In all animals, *Proteobacteria*, as facultative anaerobic organisms, commonly dominate these types of niches due to their highly flexible metabolic properties [71]. In accordance with the abundance values found in this study, the *Proteobacteria* family *Vibrionaceae* has been described to be one of the most abundant symbionts in marine fish [72, 73]. Species of the family *Vibrionaceae* are known to help with digestion through the production of chitinase, amylase, lipase, and proteases, but some of those species can also produce harmful enzymes and act as pathogens [74]. *Firmicutes* are common intestinal symbionts in fish and mammals [75, 76]. Their prevalence in gilthead sea bream intestine is high [41, 77] and their abundance is modulated by diet and age [18, 36]. *Firmicutes* contribute to the host’s nutrition by their ability to produce short-chain fatty acids and vitamins. They also have important roles in pathogen inhibition and immune training [78, 79]. The third most abundant phylum, *Actinobacteria*, contains symbionts important for the host’s health because they convert the feedstuffs into microbial biomass and fermentation products that can be utilized by the host [80]. The core bacterial composition at the family level reported here is also in agreement with other studies performed in the same species [36, 41], although not always in comparable proportions. Variations in microbiota composition within the same species can be due to many factors. Differences in environment [49, 55], season [51], age [36, 53, 54], sex [36], diet [18, 42], or genetic background [46] can difficult comparisons among studies. In addition, technical differences, such as part of the intestine sampled [55, 81], type of sample (adherent, transient, or total microbiota) [82, 83], DNA extraction techniques or analysis methodology [84, 85], can also be a source of variation. However, even though we cannot make statistically sound comparisons among studies, it is safe to say that the typical microbial architecture of gilthead sea bream intestine was found in this study and the different genetic backgrounds of the families do not alter this core composition.

Evidently, diet is a key factor in the shaping of the intestinal microbiota. It will determine the nutrient availability for the resident bacteria and thus, will benefit the growth of certain populations over others. High or total fishmeal and fish oil replacement with plant ingredients had a significant effect on gilthead sea bream intestinal microbiota decreasing the diversity and dramatically changing bacterial composition favoring the growth of certain taxa that are potential fish pathogens. This was linked with increased mortality and/or disease susceptibility [18, 41]. However, when substitution diets meet the theoretical nutrient requirements these detrimental effects can be reverted. This was the case when sodium butyrate was used as an additive in high-replacement diets in gilthead sea bream. Sodium butyrate supplementation in plant-based diets improved disease resistance to the same enteric parasite used in this study [18]. Of note, the fish used for the parasite challenge in the current study were fed a replacement diet with a less extreme formulation in order to avoid putative drawback effects related to extreme plant-based diet formulations. In this scenario, the current results show that disease resilience is also affected by the genetic background of the animals, with possible associations to changes in intestinal microbiota, known for its importance in disease resistance and immune training [86]. Fast-growth animals showed improved resistance to the intestinal parasite *E. leei*. A positive correlation between growth and disease resilience was found in other fish species, such as turbot for a parasitic ciliate [65]. However, this is not universal, as growth selection was found to be positively correlated with disease susceptibility in other fish species when facing bacterial or viral challenges [87, 88]. The parasite used in this study, *E. leei*, invades the paracellular space between the enterocytes, where it lives and divides [89], causing severe enteritis linked to the disruption of tight junctions and loss of intestinal barrier function [90]. Remarkably, even though all groups got infected, the fast-growth families showed lower parasite abundance, intensity of infection, and lower disease signs. Thus, selection for growth in gilthead sea bream seems to be linked to a selection for intestines that are able to cope better with parasitic infections. The genetic mechanisms responsible for this improved resistance remain to be elucidated.

Significant changes in intestinal microbiota were detected when fish were fed plant-based diets, but this effect was more evident when the two variables, diet, and family were considered. In mammals, it has been shown that, in controlled environments, the genetic background accounts for a substantial fraction of the abundance of most common microbiota, having direct consequences in the different responses to dietary changes [63]. Diet, genetics, and intestinal microbiota tightly interact to determine the metabolic status [91]. The current results show that the effect of the diet on intestinal microbiota is dependent on the genetic background of the animals. Fast-growth families show fewer changes at the level of bacterial composition when compared with the other two groups of families. However, these subtler changes have the capacity to account for more substantial changes at the metabolic level, whereas the significant changes detected in slow-growth families are not proportional to the few changes detected in the inferred pathway analysis. Future studies should be conducted to identify associations between specific genomic loci and microbial populations to fully define these interactions.

The changes in microbiota due to genetic background and diet, together with the differential inferred metabolic changes are reflecting, at least in part, the level of domestication of the different families. Only 30% of all farmed finfish species are considered truly domesticated and do not depend on regular inputs of wild individuals to maintain the farmed population. Gilthead sea bream is included in this 30% [92]. The results from the current study indicate that, through the domestication process of gilthead sea bream, selection for growth has been somewhat indirectly linked to plasticity to different diets and disease resistance. A similar result was described when growth trajectories and tissue and intestinal plasticity were studied in the same gilthead sea bream families. Unlike intermediate and slow-growth families, fast-growth families demonstrated the capacity to reshape their intestines to adapt to plant-based diets, with no impact on their growth parameters [31]. In animal husbandry, the presence of certain groups of bacteria has been related to improvements in feed efficiency and growth performance. For instance, in pigs, the enrichment of *Clostridiales* and microbial genes involved in fermenting dietary polysaccharides and amino acid metabolism are positively correlated with feed efficiency [93]. Similarly, prebiotics-treated fish showed improved growth performance parallel to an increased abundance of *Clostridium* spp. [94]. The present results showed a higher abundance of *Clostridiaceae* in the fast-growth families (11%) when compared with the intermediate and slow-growth families (6 and 7%, respectively). The positive effects of *Clostridiales* on growth and feed efficiency have been attributed to the production of short-chain fatty acids, with many demonstrated positive effects on animal health, including anti-inflammatory properties, epithelial barrier strengthening, and disease resistance [18, 71, 95].

The changes in the metabolic capacity of the intestinal bacteria induced by plant-based diets detected in the different families of this study are striking. Of note, we have to consider that this information was obtained from in silico inference and only reflects what could be potentially occurring, but it is still of value to assess the metabolic capability of the bacterial populations. Fast-growth families showed the potential to change the metabolic capacity of their intestinal bacteria with very subtle changes in their bacterial composition. However, the larger differences in microbial populations detected in the other families did not mirror significant changes in the pathway analysis. Moreover, opposite effects induced by plant-based diets were detected when comparing different families. For instance, the Arabinogalactan biosynthesis pathway is predicted to be highly under-represented in fast-growth families, whereas it is over-represented in the intermediate-growth family. This could be explained by the differential changes detected in the *Actinobacteria* population, which decreases in fast-growth families fed plant-based diets but increases in the intermediate-growth family. Arabinogalactan is a key component of the cell envelope of gram-positive bacteria, which constitutes the first point of contact with the host thus having an important effect on immune recognition and activation [96]. Other pathways related to infection and immune activation were inferred to be down-regulated in the fast-growth families, for instance, bacterial invasion of epithelial cells, *Staphylococcus aureus* infection, and RIG-I-like receptor signaling pathway. Interestingly, the latter was highly represented in the slow-growth families, highlighting again that the changes induced by diet in fish with different genetic backgrounds can be opposed. To clarify, these results do not imply that bacteria are expressing RIG-I-like receptor signaling pathway genes, but that some bacteria within these populations might be expressing molecules that could activate such pathway. This lower representation of immune activation pathways could indicate that these animals harbor fewer bacteria that could induce an inflammatory profile. Studies on the metatranscriptome of the different families are being conducted to reveal which bacterial genes are actually being expressed, and they will allow validation of these prediction methods.

## Conclusions

In the domestication process of aquaculture species, selective breeding programs have to consider many variables to attain robust animals with physiological plasticity to overcome changes in the culture environment, such as differences in diet or pathogenic pressures. This study demonstrated the influence of the selection for heritable growth in gilthead sea bream on intestinal bacterial populations. The genetic background, microbiota composition, and dietary and physiological plasticity are intricately linked. Despite the genomic heterogeneity, gilthead sea bream families selected for heritable growth are more robust. They adapt better to dietary changes, reshaping their intestines and organosomatic indexes, with no significant effects on their growth and health parameters [31], and can cope more efficiently with pathogens. These animals also harbor a plastic microbiota which effectively adapts to the metabolic challenges induced by dietary changes. Future studies will focus on whether and how these microbial changes correlate with health, growth, and disease resilience.

## Methods

### Ethics statement

All procedures were approved by the Ethics and Animal Welfare Committee of IATS and CSIC. They were carried out in a registered installation facility (code ES120330001055) in accordance with the principles published in the European Animal Directive (2010/63/EU) and Spanish laws (Royal Decree RD53/2013) for the protection of animals used in scientific experiments.

### Experimental design

Gilthead sea bream (*Sparus aurata*) families used in this study, belonging to the Spanish selection program of gilthead sea bream (PROGENSA), were obtained and reared as previously described [31]. Briefly, fish from families e5e2, e6e2 (fast-growth suprafamily e5e6), c2c7 (intermediate-growth family), c4c2, and e4e1 (slow-growth suprafamily c4e4) were kept in six 3000 L tanks with open flow system and natural photoperiod and temperature at the IATS facilities (Castellón, Spain: 40° 5’N; 0° 10’E). Fish were individually tagged in the dorsal muscle with passive integrated transponders (PIT) and mixed in equal proportions and with a similar number of family members in each tank. During 9 months, three tanks were fed a control diet (D1) and the other three a well-balanced plant-based diet (D2). The exact composition of the diets and details on fish rearing can be found elsewhere [31] (Additional file 7: Table S4).

For the parasite challenge, 168 fish belonging to families e5e2 (fast-growth) and c4c3 (slow-growth), with a mean bodyweight of 58 g (range 38.5–95 g), already fed with D2 for 6 months, were transported to the pathology facilities at IATS and kept mixed in the same proportion in six 500 L tanks (28 fish/tank) with open flow and natural photoperiod and temperature. All fish from this trial were fed D2 ad libitum along with the duration of the experiment. After 1 week of acclimatizing, four replicated tanks were challenged by anal intubation with the intestinal parasite *Enteromyxum leei* (0.3 ml inoculum/fish), as previously described [97]. This constituted the recipient or challenged group (R: 56 fish per family). Fish from the two remaining tanks received the same volume of PBS and constituted the control non-challenged fish (C: 28 fish per family).

### Sampling procedures

For the microbiota study, the sampling was performed in July 2018 (water temperature 22–24 °C), 9 months after the beginning of the feeding trial. Six fish from each family and diet were sampled, 2 fish from each of the triplicated tanks. This summed 12 sampled fish per diet for the fast- and slow-growth suprafamilies (e5e6 and c4e4, respectively) and 6 per diet for the intermediate-growth family (c2c7). Sampled fish were all male with a bodyweight range of 87–187 g. Animals were starved for 48 h and sacrificed by overexposure to the anesthetic 3-aminobenzoic acid ethyl ester (MS-222, 0.1 g/L). Intestines were dissected and the anterior portion was cut out, opened, and gently washed with sterile PBS to remove non-adherent bacteria. Intestinal mucus was scrapped off using the blunt edge of a sterile scalpel and collected into sterile 1.5 mL tubes. Samples were kept on ice and DNA extraction was performed immediately after the sampling. All fish were sampled in three consecutive days at the same time to avoid differences due to changes in bacterial composition in the water or temperature fluctuations.

For the parasite challenge, the sampling was performed 70 days post-infection (July 2018). All fish were starved for 48 h, sacrificed by overexposure to the anesthetic MS-222, weighted and the entire intestines were taken for parasite quantification by qPCR as previously described [62].

### DNA extraction and Illumina MiSeq sequencing of 16S rRNA amplicons

Intestinal mucus samples (200 μl) were treated with 250 μg/ml of lysozyme (Sigma) for 15 min at 37 °C. Then, DNA was extracted using the High Pure PCR Template Preparation Kit (Roche) following the manufacturer’s instructions. DNA concentration, quality, and purity were measured using a Nanodrop 2000c (Thermo Scientific) and agarose gel electrophoresis (1% w/v in Tris-EDTA buffer). DNA was stored at −20 °C until sequencing.

The V3-V4 region of the 16S rRNA gene (reference nucleotide interval 341-805 nt) was sequenced using the Illumina MiSeq system (2 × 300 paired-end run) at the Genomics Unit from the Madrid Science Park Foundation (FPCM). The details on the PCR and sequencing of amplicons are described elsewhere [36]. Raw sequence data from this experiment were uploaded to the Sequence Read Archive (SRA) under Bioproject accession number PRJNA609985 (BioSample accession numbers: SAMN14270133-192).

### Bioinformatic analysis

Raw forward and reverse reads were quality filtered using FastQC (http://www.bioinformatics.babraham.ac.uk/projects/fastqc/) and pre-processed using Prinseq [98]. Terminal N bases were trimmed in both ends and sequences with >5% of total N bases were discarded. Reads that were <150 bp long, with Phred quality score <28 in both of the sequence ends and with a Phred average quality score <26 were excluded. Then, forward and reverse reads were merged using fastq-join [99].

Taxonomy assignation was performed using the Ribosomal Database Project (RDP) release 11 as a reference database [100]. Reads were aligned with a custom-made pipeline using VSEARCH and BLAST [101, 102]. Alignment was performed establishing high stringency filters (≥90% sequence identity, ≥90% query coverage). Taxonomic assignation results were filtered and data was summarized in an Operational Taxonomic Units (OTUs) table. Sample depths were normalized by total sum scaling and then made proportional to the total sequencing depth, following the recommendations previously described [103].

### Statistical analysis

Rarefaction curves (plotting the number of observed taxonomic assignations against the number of sequences), species richness estimates, and alpha diversity indexes were obtained using the R package phyloseq [104]. Differences in species richness, diversity indexes, and phylum abundance were determined by Kruskal-Wallis test using the Dunn’s post-test, with a significance threshold of *P* < 0.05. Beta diversity across diets, groups of families, or diet × group was tested with permutational multivariate analysis of variance (PERMANOVA) using the non-parametric method *adonis* from the R package Vegan with 10000 random permutations.

To study the separation among groups (by diet or group of family), supervised partial least-squares discriminant analysis (PLS-DA) and hierarchical clustering of samples were sequentially applied using EZinfo v3.0 (Umetrics, Umea, Sweden) and R package ggplot2, respectively. Values of normalized counts of OTUs present in 5 or more samples were included in the analyses. The contribution of the different genes to the group separation was determined by the minimum Variable Importance in the Projection (VIP) values [105–107] achieving the complete clustering of the conditions, being these VIP values 1.4 in suprafamily e5e6 and 1 in family c2c7 and in suprafamily c4e4. Hotelling’s *T*^2^ statistic was calculated by the multivariate software package EZinfo v3.0. All points in the current study were within the 95% confidence limit for *T*^2^; thus, no outliers were detected and discarded. The quality of the PLS-DA model was evaluated by the parameters R2Y (cum) and Q2 (cum), which indicate the fit and prediction ability, respectively. To assess whether the supervised model was being over-fitted, a validation test consisting of 500 random permutations was performed using SIMCA-P+ (v11.0, Umetrics). Heatmap representation was constructed using the average linkage method and Euclidean distance. The same statistics were applied to rule out the tank effect.

### Metagenome prediction and pathway analysis

Piphillin was used to normalize the amplicon data by 16S rRNA gene copy number and to infer metagenomics contents [108]. This analysis was performed submitting the raw count table and the associated 16S rRNA representative sequences of the 128, 158, and 232 OTUs significantly driving the separation by diet in suprafamilies e5e6, c2c7, and c4e4, respectively (Fig. 3). For the analysis, a sequence identity cut-off of 97% was implemented, and the inferred metagenomics functions were assigned using the Kyoto Encyclopedia of Genes and Genomes database (KEGG, Oct2018 Release). Raw KEGG pathway output from Piphillin was analyzed with the Bioconductor package DESeq2 using default parameters, after flooring fractional counts to the nearest integer [36, 109, 110]. The comparisons were performed between different diets within each of the groups of families to evaluate possible pathways changing upon dietary changes. The inferred metagenomics pathways were considered differentially represented using a FDR-corrected significance threshold of 0.05.

### Parasite challenge data analysis

Quantitative parasitological variables studied were prevalence of infection (percentage of infected fish in a sampled group), mean intensity of infection (mean number of parasites per infected fish), and mean parasite abundance (mean number of parasites per fish in a sampled group, including the cero values of uninfected animals). Each individual was treated as a replicate and each group included all the fish (replicate tanks were not treated individually, as no tank effect was detected). Since *E. leei* load data in infected fish are overdispersed and aggregated, quantitative parasite load was normalized by logarithmic transformation (*y* = Log_10_(y) for intensity data and *y* = Log_10_(1 + *y*) for abundance data). Differences were assessed using the software package Prism (GraphPad). The percentage of weight gain was calculated relative to the individual weight values at the beginning of the trial. Student’s *t* test was used to determine differences in the percentage of weight gain and intensity values (normally distributed data) and Mann-Whitney and Kolmogorov-Smirnov tests to determine differences in abundance values (not normally distributed). Statistical significance was considered at *P* < 0.05.

## Supplementary information

**Additional file 1: Table S1.** Table showing the detailed sequencing data obtained in this study.

**Additional file 2: Figure S1.** Rarefaction curves obtained from the sequencing data of the 57 samples included in this study.

**Additional file 3: Figure S2.** Pie charts showing the percentage of abundance of the most abundant bacterial families (> 1% of the overall bacterial composition) in the different groups of families studied (A: e5e6, B: c2c7, C: c4e4). The color code was selected by phylum: Actinobacteria, orange; Cyanobacteria, purple; Firmicutes, blue; Proteobacteria, green; Spirochaetes, yellow; Others, grey.

**Additional file 4: Figure S3.** Goodness of fit and validations (permutation tests) of the PLS-DA models shown in this study.

**Additional file 5: Table S2.** OTUs with minimum VIP values responsible of the separation of samples by diet in the different groups of families (A: fast-growth suprafamily e5e6; B: intermediate-growth family c2c7; C: slow-growth suprafamily c4e4). VIP values represent the variable importance in projection after component 1.

**Additional file 6: Table S3.** Piphillin inferred KEGG pathways significantly different (*P*adj < 0.05) between diet groups within each group of families (A: fast-growth suprafamily e5e6; B: intermediate-growth family c2c7; C: slow-growth suprafamily c4e4). log2fc indicates the log2 fold change. SEfc is the standard error of the calculated fold change. Red and green indicate up- and down-regulated pathways in D2 relative to D1, respectively.

**Additional file 7: Table S4.** Ingredients and chemical composition of experimental diets.

## Data Availability

All relevant data are within the paper and its additional files. Raw sequence data from this experiment were uploaded to the Sequence Read Archive (SRA) under Bioproject accession number PRJNA609985 (BioSample accession numbers: SAMN14270133-192).

## Abbreviations

dpi: Days post-infection
OTU: Operational taxonomic unit
PLS-DA: Partial least squares-discriminant analysis
VIP: Variable importance in projection
