# Supplementary material for: Genetic selection for growth drives differences in intestinal microbiota composition and parasite disease resistance in gilthead sea bream
Source: Microbiome. 2020 Nov 23;8:168. doi: 10.1186/s40168-020-00922-w (PMC7686744; doi:10.1186/s40168-020-00922-w)
Supplement: Supplementary file 2 — Additional file 1: Table S1. Table showing the detailed sequencing data obtained in this study. [file 40168_2020_922_MOESM1_ESM.docx]

| **Additional file 1**. Table S1. Detailed sequencing data. | | | | | | | | |
| --- | --- | --- | --- | --- | --- | --- | --- | --- |
| **Diet** | **Samples** |  | **R1** | |  | **R2** | | **Joined and assigned reads** |
|  |  |  | **Raw reads** | **Preprocessed reads** |  | **Raw reads** | **Preprocessed reads** |  |
| D1 | c2c7-m1 |  | 277,437 | 243,645 (87.82%) |  | 277,437 | 250,226 (90.19%) | 200,111 |
| D1 | c2c7-m2 |  | 203,262 | 177,481 (87.32%) |  | 203,262 | 174,151 (85.68%) | 120,657 |
| D1 | c2c7-m3 |  | 228,762 | 198,673 (86.85%) |  | 228,762 | 196,472 (85.88%) | 135,165 |
| D1 | c2c7-m5 |  | 172,376 | 151,684 (88.00%) |  | 172,376 | 149,210 (86.56%) | 99,040 |
| D1 | c2c7-m6 |  | 200,765 | 176,772 (88.05%) |  | 200,765 | 173,115 (86.23%) | 118,599 |
| D1 | c2c7-m7 |  | 269,838 | 226,896 (84.09%) |  | 269,838 | 221,750 (82.18%) | 127,095 |
| D2 | c2c7-m1 |  | 223,216 | 194,593 (87.18%) |  | 223,216 | 194,113 (86.96%) | 135,654 |
| D2 | c2c7-m2 |  | 205,541 | 178,930 (87.05%) |  | 205,541 | 178,961 (87.07%) | 134,702 |
| D2 | c2c7-m3 |  | 224,571 | 190,907 (85.01%) |  | 224,571 | 185,292 (82.51%) | 109,604 |
| D2 | c2c7-m4 |  | 248,755 | 216,966 (87.22%) |  | 248,755 | 212,672 (85.49%) | 142,724 |
| D2 | c2c7-m7 |  | 209,470 | 176,755 (84.38%) |  | 209,470 | 174,787 (83.44%) | 109,611 |
| D2 | c2c7-m8 |  | 229,350 | 198,758 (86.66%) |  | 229,350 | 198,946 (86.74%) | 146,912 |
| D1 | c4c3-m1 |  | 306,559 | 268,269 (87.51%) |  | 306,559 | 262,350 (85.58%) | 168,740 |
| D1 | c4c3-m2 |  | 265,443 | 229,790 (86.57%) |  | 265,443 | 227,032 (85.53%) | 148,329 |
| D1 | c4c3-m3 |  | 241,158 | 219,566 (91.05%) |  | 241,158 | 219,444 (91.00%) | 188,650 |
| D1 | c4c3-m6 |  | 255,468 | 223,879 (87.63%) |  | 255,468 | 220,635 (86.37%) | 146,444 |
| D1 | c4c3-m7 |  | 256,829 | 225,818 (87.93%) |  | 256,829 | 227,161 (88.45%) | 171,260 |
| D2 | c4c3-m1 |  | 291,546 | 259,940 (89.16%) |  | 291,546 | 258,991 (88.83%) | 197,852 |
| D2 | c4c3-m2 |  | 210,819 | 183,324 (86.96%) |  | 210,819 | 179,787 (85.28%) | 122,323 |
| D2 | c4c3-m3 |  | 235,740 | 206,900 (87.77%) |  | 235,740 | 205,235 (87.06%) | 141,215 |
| D2 | c4c3-m5 |  | 229,069 | 190,801 (83.29%) |  | 229,069 | 187,395 (81.81%) | 107,106 |
| D2 | c4c3-m7 |  | 232,304 | 206,947 (89.08%) |  | 232,304 | 203,930 (87.79%) | 138,833 |
| D2 | c4c3-m8 |  | 196,993 | 173,839 (88.25%) |  | 196,993 | 170,282 (86.44%) | 111,302 |
| D1 | e4e1-m1 |  | 230,731 | 201,497 (87.33%) |  | 230,731 | 210,356 (91.17%) | 174,068 |
| D1 | e4e1-m2 |  | 236,611 | 211,809 (89.52%) |  | 236,611 | 211,472 (89.38%) | 158,095 |
| D1 | e4e1-m3 |  | 255,328 | 231,118 (90.52%) |  | 255,328 | 231,499 (90.67%) | 195,092 |
| D1 | e4e1-m4 |  | 191,896 | 166,955 (87.00%) |  | 191,896 | 164,549 (85.75%) | 106,213 |
| D1 | e4e1-m5 |  | 218,412 | 185,305 (84.84%) |  | 218,412 | 183,408 (83.97%) | 115,489 |
| D1 | e4e1-m6 |  | 263,489 | 231,124 (87.72%) |  | 263,489 | 227,676 (86.41%) | 166,990 |
| D2 | e4e1-m1 |  | 256,061 | 226,717 (88.54%) |  | 256,061 | 221,800 (86.62%) | 130,134 |
| D2 | e4e1-m2 |  | 236,839 | 207,094 (87.44%) |  | 236,839 | 208,363 (87.98%) | 165,802 |
| D2 | e4e1-m3 |  | 317,587 | 276,570 (87.08%) |  | 317,587 | 272,538 (85.82%) | 197,375 |
| D2 | e4e1-m5 |  | 228,973 | 189,906 (82.94%) |  | 228,973 | 187,526 (81.90%) | 120,191 |
| D2 | e4e1-m7 |  | 255,862 | 224,767 (87.85%) |  | 255,862 | 226,415 (88.49%) | 168,461 |
| D2 | e4e1-m8 |  | 218,884 | 194,315 (88.78%) |  | 218,884 | 190,659 (87.11%) | 133,377 |
| D1 | e5e2-m1 |  | 233,954 | 205,006 (87.63%) |  | 233,954 | 203,069 (86.80%) | 151,179 |
| D1 | e5e2-m2 |  | 336,881 | 290,795 (86.32%) |  | 336,881 | 302,051 (89.66%) | 252,332 |
| D1 | e5e2-m3 |  | 186,362 | 157,981 (84.77%) |  | 186,362 | 155,880 (83.64%) | 101,705 |
| D1 | e5e2-m4 |  | 225,802 | 191,213 (84.68%) |  | 225,802 | 193,786 (85.82%) | 151,622 |
| D1 | e5e2-m5 |  | 248,194 | 215,553 (86.85%) |  | 248,194 | 215,360 (86.77%) | 167,500 |
| D1 | e5e2-m6 |  | 288,704 | 255,958 (88.66%) |  | 288,704 | 255,755 (88.59%) | 210,362 |
| D2 | e5e2-m1 |  | 228,764 | 200,466 (87.63%) |  | 228,764 | 197,793 (86.46%) | 140,590 |
| D2 | e5e2-m2 |  | 228,633 | 200,291 (87.60%) |  | 228,633 | 197,677 (86.46%) | 133,758 |
| D2 | e5e2-m3 |  | 232,283 | 199,796 (86.01%) |  | 232,283 | 201,645 (86.81%) | 156,170 |
| D2 | e5e2-m4 |  | 239,682 | 207,053 (86.39%) |  | 239,682 | 204,424 (85.29%) | 142,608 |
| D2 | e5e2-m5 |  | 207,576 | 182,319 (87.83%) |  | 207,576 | 192,092 (92.54%) | 165,127 |
| D2 | e5e2-m6 |  | 219,911 | 188,474 (85.70%) |  | 219,911 | 187,686 (85.35%) | 138,223 |
| D1 | e6e2-m1 |  | 331,625 | 262,233 (79.08%) |  | 331,625 | 271,727 (81.94%) | 211,467 |
| D1 | e6e2-m2 |  | 263,744 | 204,322 (77.47%) |  | 263,744 | 202,457 (76.76%) | 115,077 |
| D1 | e6e2-m4 |  | 312,946 | 262,196 (83.78%) |  | 312,946 | 261,126 (83.44%) | 185,543 |
| D1 | e6e2-m5 |  | 500,581 | 394,515 (78.81%) |  | 500,581 | 401,192 (80.15%) | 303,044 |
| D2 | e6e2-m1 |  | 326,742 | 299,624 (91.70%) |  | 326,742 | 298,426 (91.33%) | 263,008 |
| D2 | e6e2-m2 |  | 208,193 | 187,145 (89.89%) |  | 208,193 | 186,001 (89.34%) | 153,239 |
| D2 | e6e2-m3 |  | 213,486 | 187,237 (87.70%) |  | 213,486 | 187,102 (87.64%) | 120,694 |
| D2 | e6e2-m4 |  | 236,727 | 210,328 (88.85%) |  | 236,727 | 211,133 (89.19%) | 171,403 |
| D2 | e6e2-m5 |  | 233,988 | 204,709 (87.49%) |  | 233,988 | 204,751 (87.50%) | 141,548 |
| D2 | e6e2-m6 |  | 236,737 | 214,409 (90.57%) |  | 236,737 | 211,768 (89.45%) | 173,788 |
