# Supplementary material for: Genetic selection for growth drives differences in intestinal microbiota composition and parasite disease resistance in gilthead sea bream
Source: Microbiome. 2020 Nov 23;8:168. doi: 10.1186/s40168-020-00922-w (PMC7686744; doi:10.1186/s40168-020-00922-w)

Additional file 2: Figure S1. Rarefaction curves obtained from the sequencing data of the 57 samples included in the study.

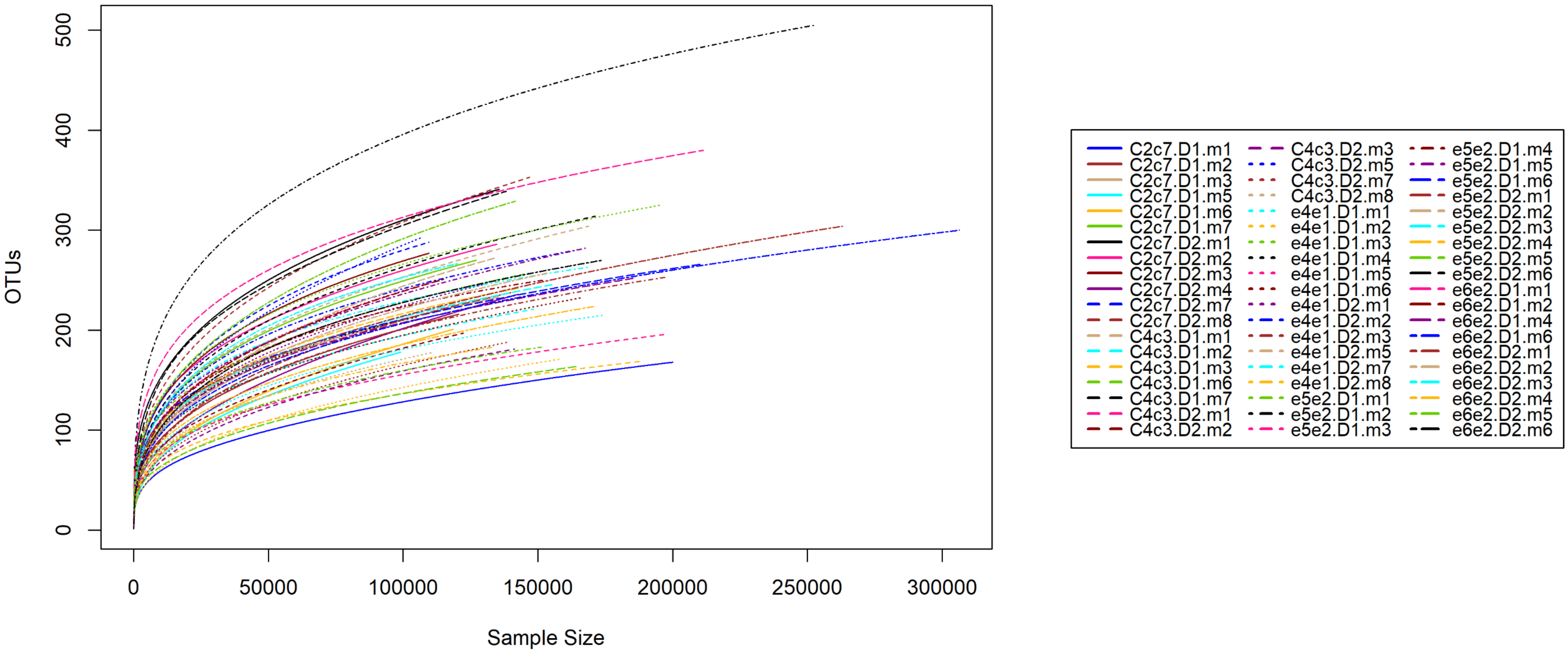

Supplement: Supplementary file 3 — Additional file 2: Figure S1. Rarefaction curves obtained from the sequencing data of the 57 samples included in this study. [file 40168_2020_922_MOESM2_ESM.pdf]
