# Supplementary material for: Genetic selection for growth drives differences in intestinal microbiota composition and parasite disease resistance in gilthead sea bream
Source: Microbiome. 2020 Nov 23;8:168. doi: 10.1186/s40168-020-00922-w (PMC7686744; doi:10.1186/s40168-020-00922-w)

Additional file 3: Figure S2. Pie charts showing the percentage of abundance of the most abundant bacterial families (> 1% of the overall bacterial composition) in the different groups of families studied (A: e5e6, B: c2c7, C: c4e4). The color code was selected by phylum: Actinobacteria, orange; Cyanobacteria, purple; Firmicutes, blue; Proteobacteria, green; Spirochaetes, yellow; Others, grey.

A

B

C

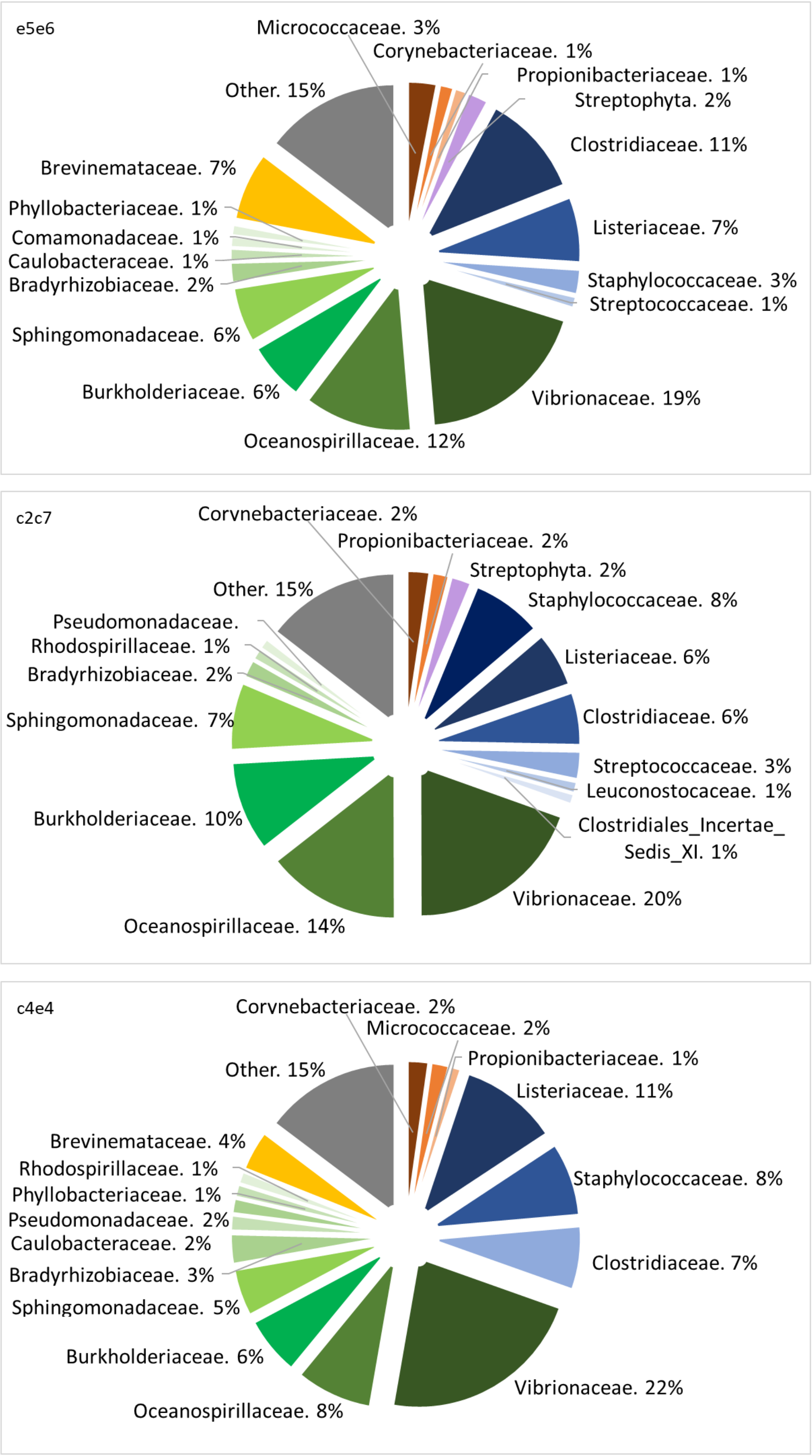

Supplement: Supplementary file 4 — Additional file 3: Figure S2. Pie charts showing the percentage of abundance of the most abundant bacterial families (> 1% of the overall bacterial composition) in the different groups of families studied (A: e5e6, B: c2c7, C: c4e4). The color code was selected by phylum: Actinobacteria, orange; Cyanobacteria, purple; Firmicutes, blue; Proteobacteria, green; Spirochaetes, yellow; Others, grey. [file 40168_2020_922_MOESM3_ESM.pdf]
