# Supplementary material for: Genetic selection for growth drives differences in intestinal microbiota composition and parasite disease resistance in gilthead sea bream
Source: Microbiome. 2020 Nov 23;8:168. doi: 10.1186/s40168-020-00922-w (PMC7686744; doi:10.1186/s40168-020-00922-w)

Additional file 4: Figure S3. Goodness of fit and validations (permutation tests) of the PLS-DA models shown in Figure 2 (A,B) and Figure 3 (C-H).

A

PLSDA model for diet. Figure 2

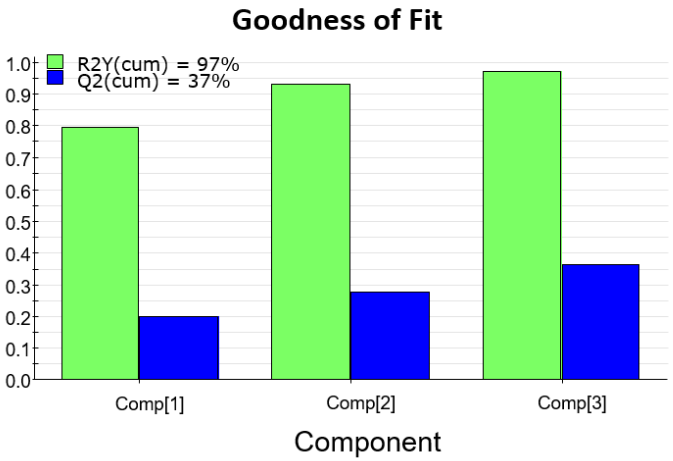

B

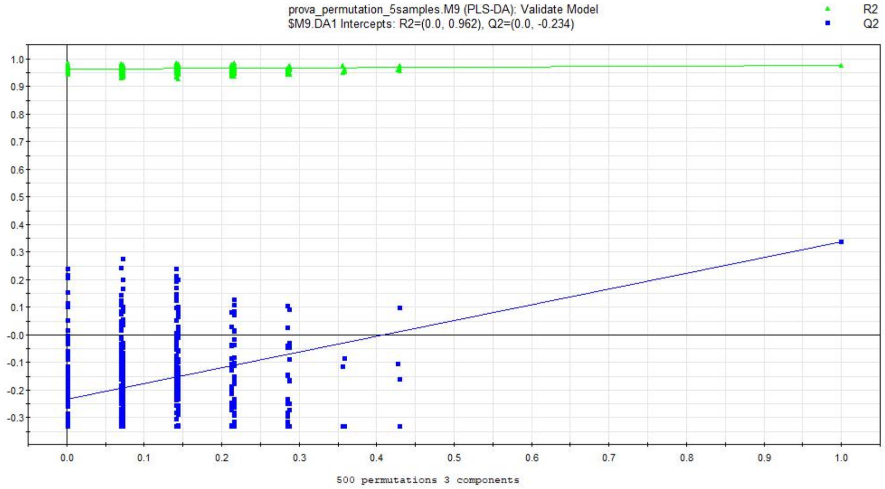

C PLSDA model for diet and suprafamily e5e6. Figure 3A

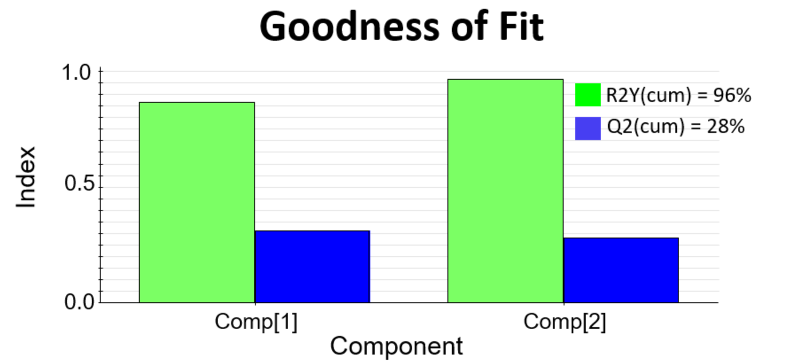

D PLSDA model for diet and family c2c7. Figure 3B

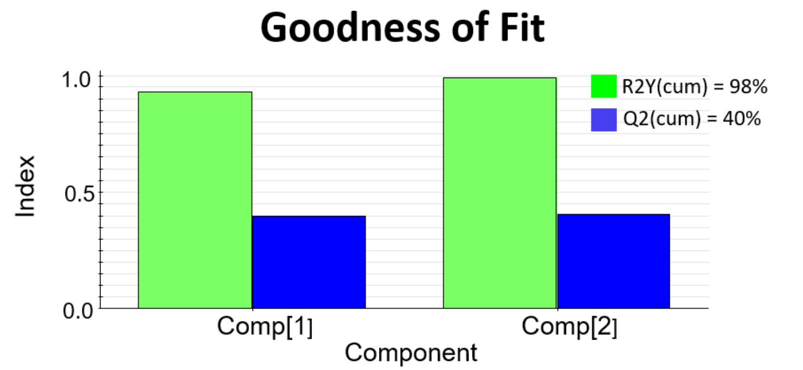

E PLSDA model for diet and suprafamily c4e4. Figure 3C

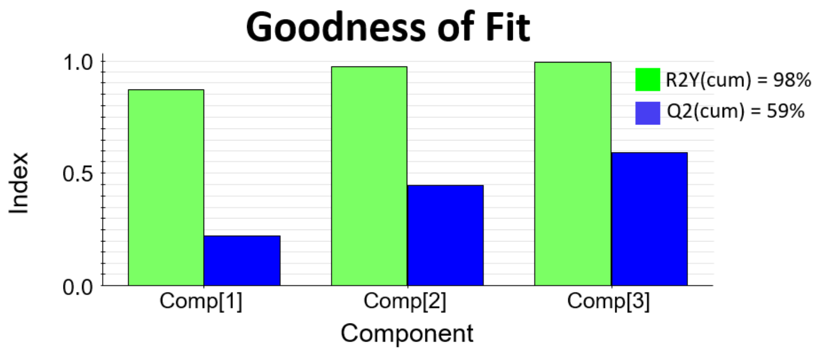

F

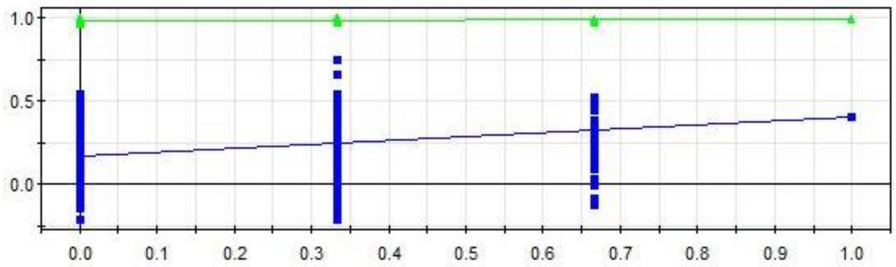

G

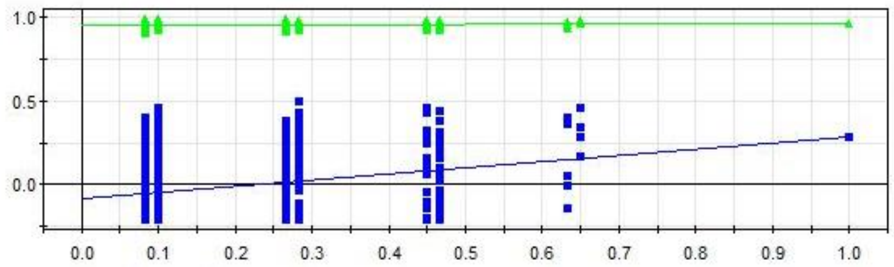

H

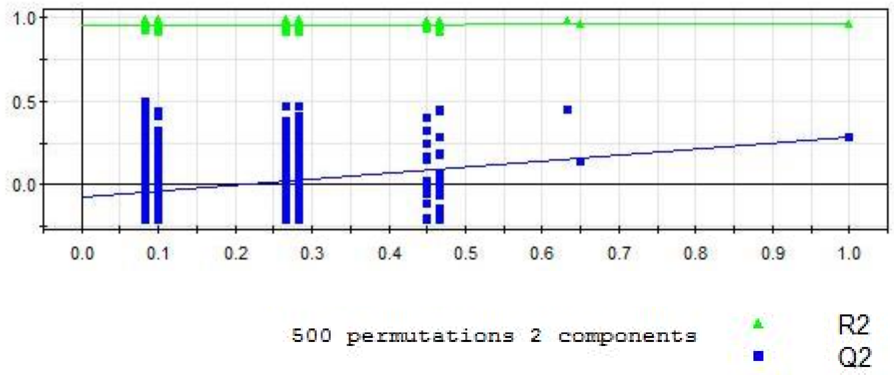

Supplement: Supplementary file 5 — Additional file 4: Figure S3. Goodness of fit and validations (permutation tests) of the PLS-DA models shown in this study. [file 40168_2020_922_MOESM4_ESM.pdf]
