# Supplementary material for: Genetic selection for growth drives differences in intestinal microbiota composition and parasite disease resistance in gilthead sea bream
Source: Microbiome. 2020 Nov 23;8:168. doi: 10.1186/s40168-020-00922-w (PMC7686744; doi:10.1186/s40168-020-00922-w)
Supplement: Supplementary file 8 — Additional file 7: Table S4. Ingredients and chemical composition of experimental diets. [file 40168_2020_922_MOESM7_ESM.docx]

**Additional file 7.** Table S4. Ingredients and chemical composition of experimental diets.

| **Ingredient (%)** | **D1** | **D2** |
| --- | --- | --- |
| Fish meal | 23.0 | 3.0 |
| Fish hydrolysate (CPSP) | 2.0 | 2.0 |
| Soya protein | 16.7 | 25.6 |
| Corn gluten | 16.5 | 25.5 |
| Wheat gluten | 4.5 | 7.3 |
| Rapeseed cake | 12.0 | 10.0 |
| Wheat | 10.0 | 7.4 |
| Fish oil | 14.1 | 3.9 |
| Rapeseed oil | 0 | 9.0 |
| Mineral-vitamin mix^a^ | 1.25 | 6.3 |
|  |  |  |
| Proximate composition (%) |  |  |
| Moisture | 7.9 | 7.5 |
| Crude protein | 45.0 | 45.0 |
| Crude fat | 20.1 | 20.1 |
| Ash | 6.9 | 5.9 |
| NFE^b^ | 19.1 | 19.8 |
| ARA^c^ | 0.17 | 0.05 |
| EPA^d^ | 2.30 | 0.60 |
| DHA^e^ | 1.50 | 0.42 |
| EPA + DHA | 3.8 | 1.02 |
| Crude energy (MJ/kg) | 22.1 | 22.3 |

^a^ Contains vitamins, minerals, amino acids, cholesterol, lecithin and anti-oxidants.

^b^ Nitrogen-free extract.

^c^ Arachidonic acid (20:4n-6).

^d^ Eicosapentaenoic acid (20:5n-3).

^e^ Docosahexaenoic acid (20:6n-3).
